# Assessment of potential candidate genes for partial resistance to Sclerotinia stem rot caused by *Sclerotinia sclerotiorum* using real‐time quantitative PCR

**DOI:** 10.1002/tpg2.20561

**Published:** 2025-02-06

**Authors:** Deus Mugabe, Mohsen Yoosefzadeh Najafabadi, Christopher Grainger, Istvan Rajcan

**Affiliations:** ^1^ Department of Plant Agriculture University of Guelph Guelph Ontario Canada

## Abstract

Sclerotinia stem rot (SSR), caused by *Sclerotinia sclerotiorum* (Lib) de Bary (*S*. *sclerotiorum*), is one of the most important diseases that causes significant soybean [*Glycine max* (L.) Merr.] seed yield and quality losses in Canada and globally. Initiation of plant defense mechanisms is crucial for establishing partial resistance to the pathogenic fungus. To understand plant response to *S*. *sclerotiorum*, we conducted a temporal (1, 3, and 5 days post‐inoculation [DPI]) assessment of gene expression changes in the stem of soybean genotypes with contrasting phenotypic response. We focused on four genes that have been previously reported as associated with SSR partial resistance and are known to be involved in defense‐related functions such as cell wall modification, signaling, response to wounding, and response to fungus. The results showed a higher and earlier expression of the genes in partially resistant cultivars compared to the susceptible. Expression of some genes increased up to 11‐ (*Glyma.02G059700*) to 16‐fold (*Glyma.09G232100*) by 3 DPI in the partially resistant cultivar, OAC Drayton, while the genes were generally downregulated in the susceptible cultivar, OAC Shire, at the same DPI. This study improves our understanding of expression patterns of genes involved in plant defense against fungal pathogens in soybean. More importantly, the knowledge of genes that are essential in defense against *S. sclerotiorum* can be used to fine‐map the quantitative trait loci for SSR resistance and facilitate accelerated breeding of SSR‐resistant cultivars through gene‐based marker‐assisted selection.

## Core Ideas

- Importance of Sclerotinia stem rot (SSR) as a major soybean disease globally, with significant yield and quality losses.
- Accelerated initiation of plant defense mechanisms is crucial for partial resistance to SSR.
- Temporal assessment of gene expression changes in soybean stems with varying levels of resistance to SSR.
- Four genes associated with SSR resistance showed higher expression in partially resistant cultivars.
- Potential for development of SSR‐resistant soybean cultivars through gene‐based marker‐assisted selection for SSR.

**Abbreviations**
DPI: days post‐inoculation
GWAS: genome‐wide association study
qRT‐PCR: real‐time quantitative reverse transcription polymerase chain reaction
QTL: quantitative trait locus
SSR: Sclerotinia stem rot

## 1 INTRODUCTION

*Sclerotinia sclerotiorum* (Lib) de Bary (*S*. *sclerotiorum*) is a fungal pathogen that affects soybeans [*Glycine max* (L.) Merr.], leading to the development of white mold or Sclerotinia stem rot (SSR) disease (Boland & Hall, 1987). Additionally, the pathogen infects a wide range of plants spanning 408 species, primarily dicotyledons, although infections have been reported in monocotyledon species (Boland & Hall, 1987). *S. sclerotiorum* is known to thrive in highly humid environmental conditions with ∼25°C temperatures (Moellers et al., 2017; Rousseau et al., 2004; Yoosefzadeh‐Najafabadi & Rajcan, 2022). In soybeans, the fungus largely infects the flower and proliferates through the main stem, leading to bleaching, shredding of tissue, and wilting (Bolton et al., 2006). Since 1978, the prevalence of SSR outbreaks has increased significantly across the globe leading to yield losses of up to 94% in extreme SSR outbreak seasons, including in major soybean‐growing regions of the Northern United States and Canada (Boland & Hall, 1987; Cline & Jacobsen, 1983; Z. Wang et al., 2019). Additionally, soybean seed quality is impacted by SSR through impacting oil content and reduced plant germination (Hoffman et al., 1998; Mueller et al., 2002).

SSR is very challenging to control in soybeans due to its sensitivity to environmental conditions and the levels of *S. sclerotiorum* in soybean‐growing fields (Peltier et al., 2012). The environmental sensitivity leads to high variability of disease incidence and severity from year to year, which makes it challenging to plan and deploy useful in‐season control methods (Peltier et al., 2012). Various agronomic and chemical measures, including crop rotation, reduced tillage, wide row spacing, and fungicide application, have been evaluated for controlling SSR in soybean production but have proven both costly and ineffective (Mueller et al., 2002; Peltier et al., 2012).

Genetic resistance is the most effective, economical, and environmentally friendly method to control diseases in crops and is regarded as the most viable option to manage SSR (Kurle et al., 2001; Moellers et al., 2017). Although no complete resistance to SSR has been found, sources of partial resistance have been found in some soybean genotypes (Grau & Gillespie, 1982; Hoffman et al., 2002). Deciphering the genetics underlying SSR partial resistance is difficult due to its quantitative nature. In soybeans, SSR partial resistance is governed by many genes contributing small effects and is easily influenced by the environment, genotype, and interaction between environment and genotype (X. Zhao et al., 2015). Several studies have used field, laboratory, and controlled environment inoculation methods in combination with genetic mapping tools to unravel the genetics that control resistance to SSR resistance (Bastien et al., 2014; Boudhrioua et al., 2020; Grau & Bissonnette, 1974; Moellers et al., 2017; Wei et al., 2017). Through biparental linkage mapping, studies have reported over 100 quantitative trait loci (QTLs) that control SSR partial resistance on 18 out of 20 soybean chromosomes, which have been recorded on SoyBase, the USDA‐ARS Soybean Genetics and Genomics Database (https://www.soybase.org/, accessed on February 15, 2024). Due to the drawbacks of limited genetic diversity in biparental linkage mapping, genome‐wide association studies (GWAS) have been conducted to discover QTL for SSR partial resistance in populations of unrelated genotypes (Boudhrioua et al., 2020; Moellers et al., 2017; Wei et al., 2017). As a result, over 130 marker‐trait associations for SSR partial resistance have been reported in different soybean populations (Boudhrioua et al., 2020; Moellers et al., 2017; Wei et al., 2017).

Discerning the true physiological resistance to SSR is confounded by numerous factors that may lead to disease escape such as flowering time, plant density, canopy architecture, and maturity (Kim et al., 1999; Nelson et al., 1991). This complexity presents challenges in determining whether the QTL discovered through mapping studies are linked to these escape mechanisms or are associated with physiological resistance (Kandel et al., 2018; Kim et al., 1999; Rousseau et al., 2004). Reported physiological changes associated with partial resistance to SSR in soybean include the reprogramming of the phenylpropanoid pathway, resulting in elevated antifungal activities (Ranjan et al., 2019). Significant innovations in platforms for genomic and transcriptomic research have been made in recent decades, providing scientists with powerful tools for investigating different biological processes such as plant–pathogen molecular interactions, gene expression, and functional analyses (Deepak et al., 2007; Hesami et al., 2023; Razzaq et al., 2021).

Various technologies, including RNA‐sequencing (RNA‐Seq), that can detect both known and novel gene transcripts, and real‐time quantitative reverse transcription polymerase chain reaction (qRT‐PCR), primarily utilized for quantifying the expression of genes with known sequences, are presently used to unveil important insights into the transcripts associated with disease resistance in plants (Deepak et al., 2007). Notably, fluorescence‐based qRT‐PCR has become one of the most adopted methods for gene expression research, especially when studying a few genes (Abdallah & Bauer, 2016). Its widespread use is attributed to its ability to meet assay preconditions that lead to reliable results (Abdallah & Bauer, 2016). This includes the assay's demonstration of sensitivity, allowing the detection of subtle changes in gene expression; quantitativeness, providing accurate measurements of gene expression levels; and specificity, ensuring that the assay targets the gene of interest with precision (Abdallah & Bauer, 2016). In addition, the fluorescence‐based qRT‐PCR assay provides reproducibility across different biological replicates and consistency in different laboratories (Abdallah & Bauer, 2016).

For breeders to adopt genetic markers, they require assurance of both effectiveness and reliability (Yoosefzadeh‐Najafabadi, Hesami et al., 2023). The development of reliable markers faces technical limitations, especially in the discovery of QTL, where errors in phenotyping, genotyping, and computational procedures can lead to false QTL discoveries (H. Hong et al., 2022; Tibbs Cortes et al., 2021; van den Oord & Sullivan, 2003; Yoosefzadeh‐Najafabadi et al., 2023; K. Zhao et al., 2007). Conducting studies that extend beyond QTL discovery to assess their validity is a crucial step that can help address this issue (Gyawali et al., 2019; Takagi et al., 2013; Varshney et al., 2008). However, such efforts are hindered by various factors, including systemic flaws in the research realm that prioritize studies with novel QTL discoveries over replication or validation studies. This issue was further highlighted by Rajsic et al. (2023), who surveyed 24 genomic journals and found that higher ranked journals often prioritize new studies on QTL identification over replication or validation of previous research findings.

Currently, there is a lack of investigations to follow up on discovered QTL after conducting GWAS for SSR resistance in soybeans. Gene expression analysis can provide useful information about post‐infection response and genetic mechanisms underlying partial resistance to *S. sclerotiorum* in soybean. Therefore, the objective of this study is to study the expression of selected candidate genes: *Glyma.02G059400*, *Glyma.02G059700*, *Glyma.09G232100*, and *Glyma.09G232600*. The candidate genes were discovered through GWAS and gene ontology (GO) analysis by Mugabe et al. (2024) and selected for their involvement in defense against *S. sclerotiorum* in soybean. Partially resistant cultivars OAC Drayton and OAC Petrel, and susceptible cultivars, Nattosan and OAC Shire, were inoculated, and qRT‐PCR was used for gene expression analysis at 1–5 days post‐inoculation (DPI). Our results show a sharp but transient upregulation of all four genes at 1 DPI in a partially resistant soybean cultivar, OAC Drayton.

**Core Ideas**

- Importance of Sclerotinia stem rot (SSR) as a major soybean disease globally, with significant yield and quality losses.
- Accelerated initiation of plant defense mechanisms is crucial for partial resistance to SSR.
- Temporal assessment of gene expression changes in soybean stems with varying levels of resistance to SSR.
- Four genes associated with SSR resistance showed higher expression in partially resistant cultivars.
- Potential for development of SSR‐resistant soybean cultivars through gene‐based marker‐assisted selection for SSR.

## 2 MATERIALS AND METHODS

### 2.1 Plant material and inoculation

Four cultivars with differential phenotypic response to *S. sclerotiorum* were used for gene expression analysis. OAC Drayton and OAC Petrel were selected as the most partially resistant (PR) cultivars, while Nattosan and OAC Shire were selected as the most susceptible (S) cultivars based on phenotyping results of a soybean association mapping study by Mugabe et al. (2024). The selected cultivars were inoculated and phenotyped again for the current study. For inoculation, plants were grown in a randomized complete block design with three replicates. At the beginning of flowering, plants were inoculated using the cotton pad method, as described by Bastien et al. (2012). The NB‐5 strain of *S. sclerotiorum*, acquired from Dr. Francois Belzile at Laval University, Quebec, Canada, was used for the experiment. An uninoculated control group of the same cultivars was included alongside the inoculated treatment group in the study. Disease resistance was measured as lesion length in mm on the plant stem using a digital caliper (Mastercraft). Full details regarding the entire phenotyping process have been published in Mugabe et al. (2024).

### 2.2 RNA extraction and cDNA synthesis

The RNA isolation process began by harvesting 3 cm of stem tissue nearest to the inoculated flower at 1, 3, and 5 DPI. Each tissue sample was immediately frozen in liquid nitrogen upon harvest. Harvested tissue samples were then ground into a fine powder in a mortar and pestle and stored in microfuge tubes at −80°C for RNA extraction. RNA extractions were conducted using the PurelinkTM RNA Mini Kit (Invitrogen), while genomic DNA contamination was removed using the On‐column Purelink DNAse treatment (Invitrogen). The quantity and quality of the total RNA extracted was determined using the Nanodrop ND 1000 Spectrophotometer (Nanodrop Technologies, Inc.).

For cDNA synthesis, RNA sample concentrations were standardized to 200 ng/µL prior to transcription and reverse transcribed to cDNA with iScript cDNA Synthesis Kit (BioRad Laboratories) according to the manufacturer's protocol. PCR was then performed using the Eppendorf MasterCycler Pro Thermal Cycler (Merck Group) with the following protocol: 95°C for 1 min, followed by 35 cycles of a denaturing step of 95°C for 15 s, an annealing step of 51°C for 15 s, and an extension step of 72°C for 1 min. The protocol finished with a final extension step of 72°C for 10 min.

### 2.3 Candidate gene selection and primer design

Candidate gene selection was based on GWAS for SSR resistance in results presented on soybeans by Mugabe et al. (2024). In the study, two QTLs (one on chromosome 2 and one on chromosome 9) were found to be significantly associated with SSR resistance in soybeans. Subsequently, up to 62 candidate genes (32 on chromosome 2; 30 on chromosome 9) were found in the estimated linkage disequilibrium (LD) distance of 100 kb, surrounding the significant peak single nucleotide polymorphism for each QTL. However, only four candidate genes were selected based on gene role in plant defense as presented by GO annotation and the GO term enrichment report on the soybase.org database and previous studies (Table 1). Exon/intron boundaries of selected candidate genes were determined by aligning each cDNA sequence with the corresponding genomic sequence and downloaded from the Phytozome plant comparative genomics database (https://phytozome‐next.jgi.doe.gov/, accessed on August 3, 2023). Primers were designed through the online PCR primer design software Primer3plus (http://www.primer3plus.com/, accessed on August 10, 2023) with melting temperatures of 52°C–60°C, primer lengths of 20–25 bp, and amplicon lengths of 140–194 bp (Table 1). Glyma05g29000.1 (TUA5 ‐ α‐tubulin) (Table 1) was selected as the endogenous control gene in this study based on previously published results (Q. Wan et al., 2017). Primer sets for all genes were synthesized by Agriculture and Food Laboratory Services at the University of Guelph (afl.uoguelph.ca, accessed on August 10, 2023).

**TABLE 1:** List of genes, their respective primer sequences, and related information.

Gene	Purpose	GO biological process description	Forward primer (5′→3′)	Reverse primer (5′→3′)	Amplicon length	Reference
*Glyma.02G059400*	Target	Carbohydrate biosynthetic process; jasmonic acid biosynthetic process; response to fungus; response to jasmonic acid stimulus; response to wounding	CCACATCGCTCAACACCAAA	AATGACCCTCGACGTTTCCT	191	(Khoei et al., 2021)
*Glyma.02G059700*	Target	Cellular response to chitin; cellular response to molecule of bacterial origin; innate immune response	GTTGCCAAGCTTACCTCACC	CGAGGTTGAAAGAGGGGACT	194	(Severin et al., 2010)
*Glyma.09G232100*	Target	Cell wall modification; glucuronoxylan metabolic process; plant‐type cell wall biogenesis; xylan biosynthetic process	CCCGAGCCATGCAGATACTA	TTCCATCTAAACCGCACCCT	190	(J. Wang et al., 2020)
*Glyma.09G232600*	Target	Chloroplast relocation; defense response to bacterium; photosynthetic electron transport in photosystem I; photosystem II assembly; positive regulation of transcription, DNA‐dependent; rRNA processing; regulation of protein dephosphorylation; thylakoid membrane organization; transcription from plastid promoter	TTGGATCATCGGACACTGCT	GCTTTTCTTCTTGCCTGGCT	141	(Lanubile et al., 2015)
*Glyma05g29000.1* (*TUA5‐α‐tubulin*)	Normalization	N/A	AGGTCGGAAACTCCTGCTGG	AGGTGTTGAAGGCGTCGTG	159	(Q. Wan et al., 2017)

Abbreviation: GO, gene ontology.

### 2.4 Real‐time quantitative reverse transcription polymerase chain reaction

QRT‐PCR runs were performed with QuantStudio 6 Flex System (Life Technologies Inc. by Thermo Fisher Scientific). Three biological replicates and three technical replicates were prepared for each sample assay and ran on 96‐well plates. Each plate included (i) treatment assays from inoculated plants, (ii) control assays from uninoculated plants, (iii) endogenous control gene assays, and (iv) sterilized nuclease‐free water (New England Bio Labs) for negative control. The total volume for each reaction assay was 20 µL containing 10 µL of Platinum SYBR Green qRT‐PCR SuperMix‐UDG (Invitrogen), 0.8 µL (5 µM) of each forward and reverse primer, 1 µL of template genomic cDNA (40 ng), and 7.4 µL of sterilized nuclease‐free water (New England Bio Labs). Cycling parameters included an initial hold at 50°C and a subsequent hold at 95°C, each for 2 min, and 40 cycles of 95°C for 15 s and 60°C for 60 s. Data were captured during each cycle and acquired post‐read using the QuantStudio software.

### 2.5 Statistical analysis

Comparative gene expression analysis was performed by calculating and comparing fold changes (Fcs) in gene expression following the Livak and Schmittgen (2001) 2^−(ΔΔCt)^ method as follows:

(1) $$\Delta \mathrm{Ct}\;=Ct_{\mathrm{GOI}}-Ct_{\mathrm{RefG}}$$

(2) $$\Delta \Delta \mathrm{Ct}\;=\;\Delta \mathrm{Ct}\;-\Delta Ct_{\mathrm{Control}}$$

(3) $$\mathrm{Fold}\;\mathrm{change}\;=2^{-\left(\Delta \Delta \mathrm{Ct}\right)}$$

where ΔCt is the difference in the qRT‐PCR cycle critical threshold (Ct) averages of the gene of interest (Ct_GOI_) and reference gene (Ct_RefG_), ΔCt_Control_ is the average of the ΔCt of the control samples, and ΔΔCt is the difference in the ΔCt and ΔCt_Control_ values.

Analysis of variance (ANOVA), followed by Tukey's honestly significant difference (HSD) test for multiple pairwise comparisons, was used to calculate mean differences of Fcs (*α* ≤0.05). The subtraction and exponential calculations in the Livak and Schmittgen (2001) method for comparative gene expression analyses were performed using MS Excel 2016 Version 16.0 (Microsoft), while ANOVA and Tukey's HSD tests were conducted with R Studio statistical package (RStudio Team, 2020) and AllInOne packages version 1.9.5 (Yoosefzadeh‐Najafabadi et al., 2023) in R version 4.3.2.

## 3 RESULTS

### 3.1 Phenotypic response of experimental cultivars to SSR inoculation

**FIGURE 1:**
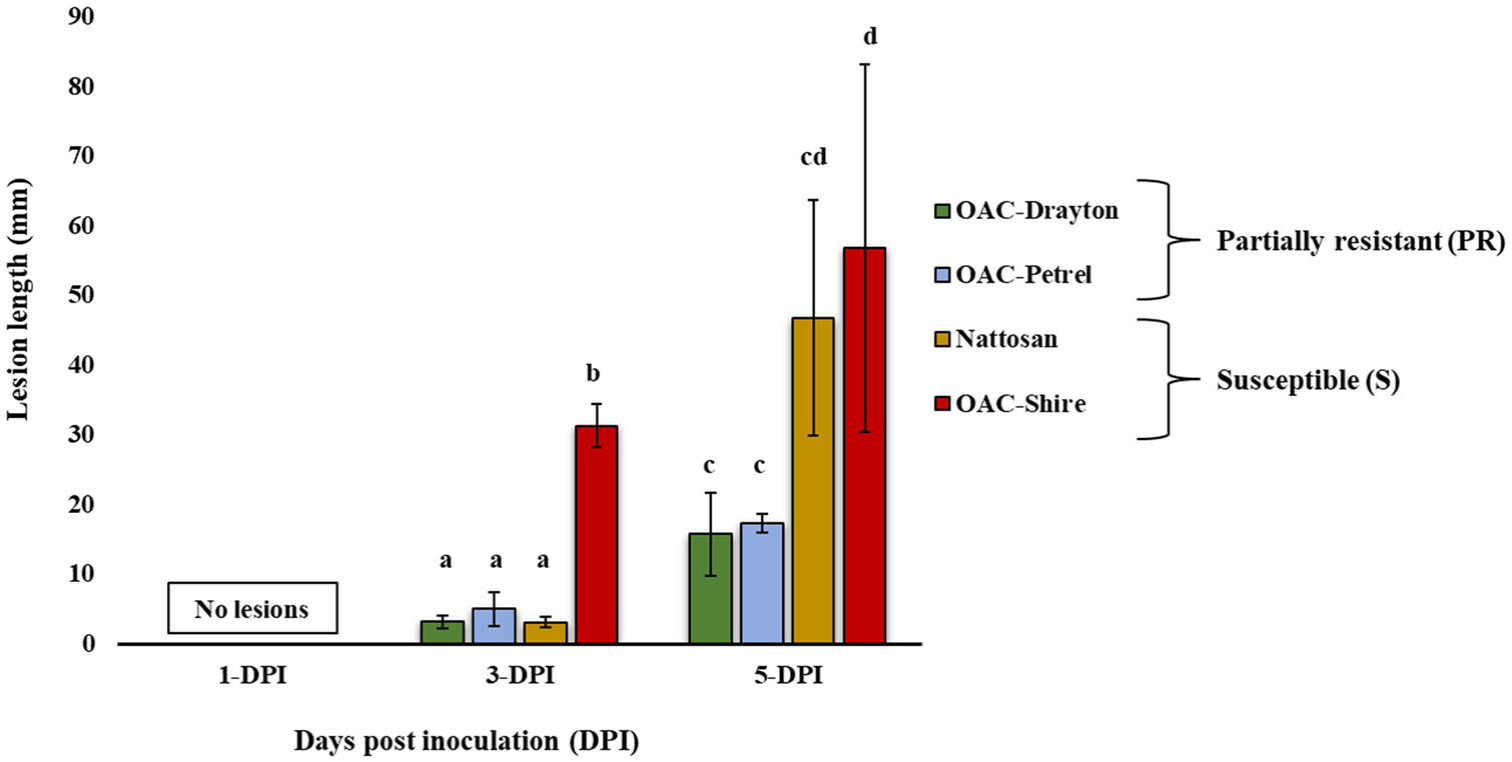
Lesion lengths of the four lines for real‐time quantitative reverse transcription polymerase chain reaction (qRT‐PCR) after inoculation with *Sclerotinia sclerotiorum*. Bars with different letters indicate significantly different lesion lengths (*p* < 0.05) at each time point. No lesions were observed at 1 day post inoculation (DPI).

While the four cultivars selected for the current study were chosen based on their known PR or S phenotypic responses to SSR, lesion length was measured on inoculated plants at three different timepoints (1, 3, and 5 DPI) of the experiment (Figure 1). Results revealed no lesion development at the first time point (1 DPI). However, a mixed response of lesion development was observed at 3 DPI due to delayed lesion development of Nattosan, while all cultivars showed expected phenotypic responses to SSR based on the resistance.

Although the mean lesion was not significantly different (*p* > 0.05) between cultivars at 3 DPI, shorter mean lesions were observed on the partially resistant OAC Drayton (3 ± 0.8 mm) and OAC Petrel (5 ± 2.4 mm) compared to S cultivars Nattosan and OAC Shire (Figure 1). Nattosan also showed a short mean lesion of 3.1 ± 0.78 mm, while OAC Shire had the longest mean lesion of 31.3 ± 3.1 mm at 3 DPI (Figure 1). Significant differences in mean lesions were found at 5 DPI (*p* < 0.05). PR cultivars OAC Drayton and OAC Petrel had the shortest mean lesions of 15.8 ± 5.9 mm and 17.3 ± 1.3 mm, respectively, whereas S cultivars, Nattosan and OAC Shire, had longer mean lesions at 46.8 ± 16.9 mm and 56.8 ± 26.4 mm, respectively (Figure 1).

### 3.2 Relative gene expression

**FIGURE 2:**
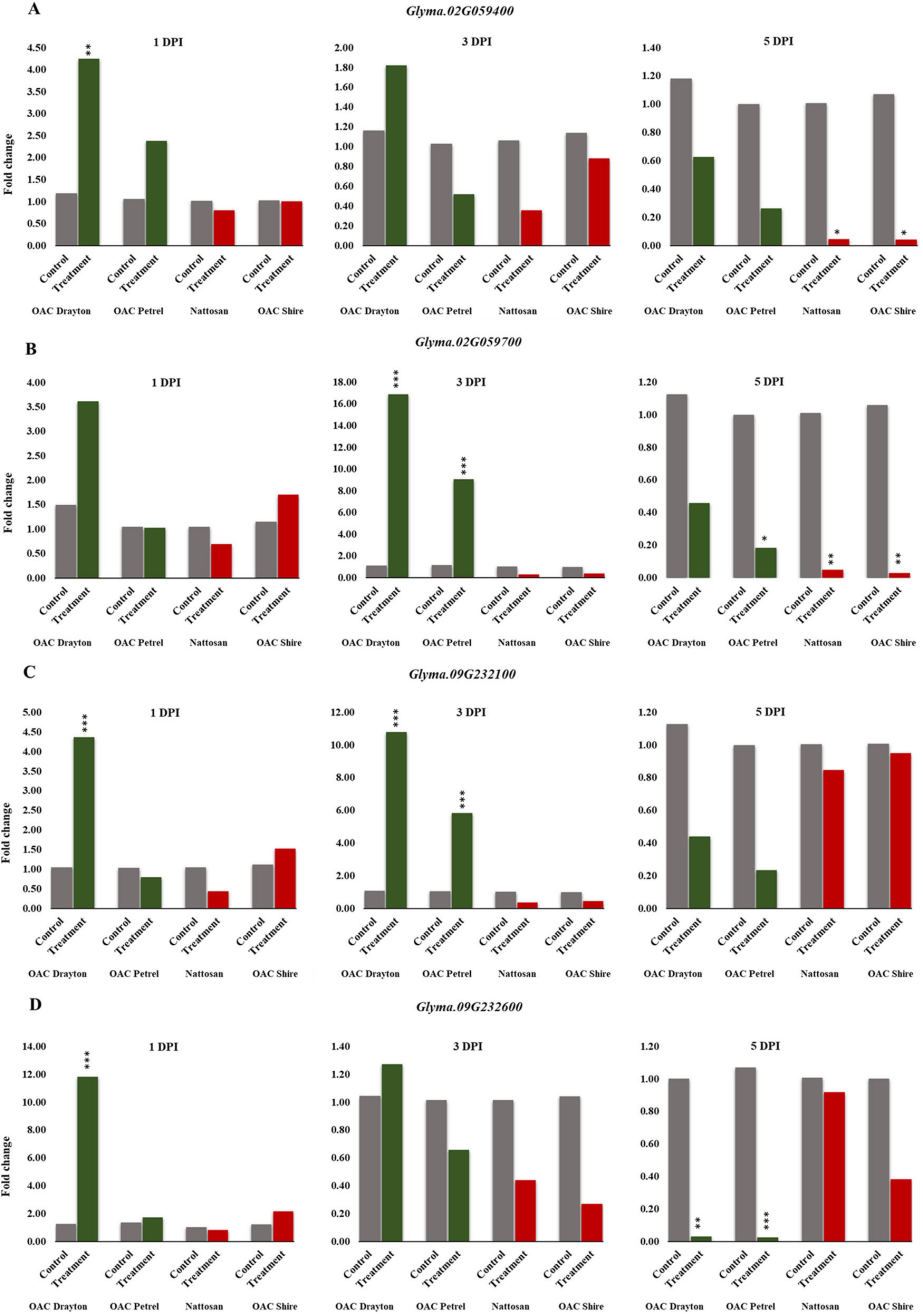
Relative quantification of *Glyma.02G059400* (A), *Glyma.02G059700* (B), *Glyma.09G232100* (C), and *Glyma.09G232600* (D) at three time points. The *Y*‐axis represents relative expression values in fold change, which were derived from comparing the expression of genes at each timepoint of inoculated versus control using the 2^−(ΔΔCt)^ method after normalization with the TUA5 reference gene. Fold change significance level is indicated as ***0.001, **0.01, and *0.05.

Gene expression analysis revealed consistent trends and significant transcriptional changes in all four target genes under the treatment condition. Overall, there was a substantial upregulation observed only in the PR cultivars at DPIs, while significant downregulation or no expression changes were generally observed for the S cultivars throughout the DPIs (Table 2; Figure 2).

**TABLE 2:** Cycle thresholds (CT) means and standard deviation (SD) means obtained from stem tissues of all cultivars under treatment and control conditions.

		1 DPI				3 DPI				5 DPI			
		Control		Treatment		Control		Treatment		Control		Treatment	
	Genotype	Mean CT	SD	Mean CT	SD	Mean CT	SD	Mean CT	SD	Mean CT	SD	Mean CT	SD
***Glyma.02G059400***	OAC Drayton	28.67	1.10	27.57	0.52	29.11	1.10	28.27	0.46	28.38	1.01	29.22	0.48
	OACPetrel	28.58	0.78	28.30	0.73	27.68	0.43	28.63	0.56	27.71	0.45	29.79	0.54
	Nattosan	27.61	0.43	28.11	1.13	26.67	0.68	27.62	0.29	26.97	0.30	29.46	0.59
	OAC Shire	27.83	0.49	27.72	0.39	28.52	0.85	28.53	1.12	27.46	0.67	30.17	0.77
***Glyma.02G059700***	OAC Drayton	28.02	1.61	26.65	0.51	28.25	0.74	28.28	0.27	28.35	1.10	28.98	0.24
	OACPetrel	27.25	0.52	27.68	0.46	27.95	0.98	30.12	0.60	27.08	0.18	29.16	0.70
	Nattosan	26.13	0.65	27.27	0.94	26.87	0.32	28.29	0.58	26.62	0.24	28.94	0.58
	OAC Shire	27.24	0.88	26.92	0.48	27.63	0.18	28.89	1.08	26.82	0.68	30.06	1.23
***Glyma.09G232100***	OAC Drayton	28.53	1.41	26.75	0.48	27.82	0.69	28.02	0.14	27.46	0.82	28.50	0.24
	OACPetrel	27.59	0.54	30.31	1.20	27.35	0.63	28.70	0.67	26.57	0.16	28.65	0.67
	Nattosan	26.76	0.82	28.99	2.60	26.68	0.36	27.82	0.39	26.11	0.22	28.50	0.30
	OAC Shire	27.57	0.79	27.56	0.68	27.27	0.28	28.55	1.28	26.71	0.24	28.99	0.53
***Glyma.09G232600***	OAC Drayton	28.36	1.77	25.75	0.15	26.99	0.46	27.24	0.28	26.63	0.56	28.40	1.00
	OAC Petrel	26.17	0.59	26.37	0.45	26.72	0.27	28.72	3.70	26.06	0.61	28.02	0.44
	Nattosan	25.96	0.49	26.24	0.43	26.27	0.28	27.06	0.34	26.17	0.23	28.09	0.53
	**OAC Shire**	27.04	1.10	25.95	0.14	27.31	4.38	27.84	1.33	25.85	0.10	29.06	0.58

Abbreviation: DPI, days post inoculation.

Notably, significantly increased relative expression for all four genes was observed in the most PR cultivar, OAC Drayton, during the early to mid‐stages of the pathogen interaction (Table 2; Figure 2). Calculated Fcs indicated significant alterations (*p* < 0.05) in gene expression, ranging from a fourfold (*Glyma.02G059400* and *Glyma.09G232100*), threefold (*Glyma.02G059700*), and up to a 12‐fold (*Glyma.09G232600*) increase in the expression for PR OAC Drayton at 1 DPI (Table 2; Figure 2). The genes exhibited a similar trend for OAC Drayton at 3 DPI, with *Glyma.02G059700* and *Glyma.09G232100* showing a 17‐ and 11‐fold increase in expression, respectively. Although the Fc in gene expression was elevated for *Glyma.02G059400* and *Glyma.09G232600* at 3 DPI in OAC Drayton, the increase was not significant (*p* > 0.05) (Table 2; Figure 2).

At 1 DPI, only *Glyma.02G059400* was upregulated for OAC Petrel, though not significantly (Fc: 2.4, *p* > 0.05). *Glyma.02G059700*, *Glyma.09G232100*, and *Glyma.09G232600* indicated either no change or a nonsignificant change in expression levels at this time point for the genotype (Table 2; Figure 2). At 3 DPI, *Glyma.02G059700* and *Glyma.09G232100* were significantly upregulated for OAC Petrel (Fc increase of 9 and 6, respectively, *p* < 0.05). Although *Glyma.02G059400* and *Glyma.09G232600* were found to be downregulated for OAC Petrel at 3 DPI, the drop in their expression was not significant (*p* > 0.05) (Table 2; Figure 2).

A relative downregulation for all genes was observed across all cultivars under the treatment condition at 5 DPI. The downregulation was more pronounced for *Glyma.02G059400* and *Glyma.02G059700* in the susceptible group (Nattosan and OAC Shire), where the expression levels dropped by approximately 96% relative to the control for both genes (*p* < 0.05). Interestingly, a significant downregulation (*p* < 0.05) was observed for *Glyma.09G232600* in the PR group, where the expression level decreased by 97% for OAC Drayton and 98% for OAC Petrel at this time point (Table 2; Figure 2).

## 4 DISCUSSION

*S. sclerotiorum* is known to have a wide range of arsenal including secreted effectors, cell wall‐degrading enzymes, detoxification enzymes, and metabolites, that it employs to successfully attack its many hosts including soybean (Bolton et al., 2006). As a result, partial resistance to the pathogen in soybean is intricately governed by many genes with varying effects (X. Zhao et al., 2015). In this study, we aimed to unravel gene expression patterns of four defense‐related genes (*Glyma.02G059400*, *Glyma.02G059700*, *Glyma.09G232100*, *Glyma.09G232600*) between PR and S cultivars in response to early SSR exposure.

During the inoculation experiments, plants showed lesions with varying lengths at 3 DPI. Interestingly, the S cultivar, Nattosan, showed statistically similar lesion length with R cultivars at 3 DPI, while the other S cultivar, OAC‐Shire, showed a significantly longer lesion length (Figure 1). The cause for the later lesion development on Nattosan is not known. By 5 DPI, the PR cultivars, OAC Drayton and OAC Petrel, had shorter lesions compared to the significantly longer lesions of the S cultivars, Nattosan and OAC Shire (*p* < 0.05; Figure 1). Confirming the contrasting phenotypic response of the selected lines was fundamental for investigating the selected genes’ involvement in providing enhanced resistance against the *S. sclerotiorum* pathogen. Tissue samples for analysis were collected on the stem at the inoculation site for the three time points (1, 3, and 5 DPI). Most changes in expression levels of the genes across the response groups occurred at 3 DPI or later, which is consistent with previous reports on differential gene expression changes after *S. sclerotiorum* infection (McCaghey et al., 2019; Westrick et al., 2019).

The expression levels of *Glyma.02G059400* were significantly higher in PR lines (Fc > 2; *p* < 0.05) compared to S cultivars as early as 1 DPI (Table 2; Figure 2). Through GO enrichment and biological function analysis, *Glyma.02G059400* has been reported to play roles in response to wounding, carbohydrate biosynthesis, and, very importantly, jasmonic acid biosynthesis. Jasmonic acid (JA) is a phytohormone with a broad range of functions in plant physiological processes. However, it has a primary function as a signal mediator against herbivorous insects and necrotrophic pathogens, where *S. sclerotiorum* is categorized. During plant defense, JA helps trigger the expression of pathogenesis‐related genes and regulates secondary metabolism, promoting the production of antipathogenic compounds (Macioszek et al., 2023). Given *S. sclerotiorum*’s ability to upregulate host reactive oxygen species (ROS) levels through oxalic acid and induce programmed cell death for pathogenic success (Kim et al., 1999; McCaghey et al., 2019; Ranjan et al., 2019), rapid mobilization of the genes involved in JA biosynthesis, including *Glyma.02G059400*, can be critical for enhanced resistance to *S. sclerotiorum*.

An early expression increase was also observed for *Glyma.02G059700* in PR lines compared to the S lines (Fc > 2, *p* < 0.05). Out of the four genes tested, *Glyma.02G059700* had the steepest Fc throughout the study, with OAC Drayton showing a 17‐fold increase, while OAC Petrel indicated a ninefold increase in the gene's expression at 3 DPI (Table 2; Figure 2). Despite the downregulation of the gene across all cultivars at 5 DPI, results suggest increased resistance to *S. sclerotiorum*, with higher expression of *Glyma.02G059700* in PR versus S cultivars. This was indicated by the observed inverse relationship between *Glyma.02G059700*’s expression levels and genotype lesion lengths at 5 DPI. The main functions of *Glyma.02G059700* described through gene annotation, GO enrichment, and biological function analysis include cellular response to chitin and innate immune response through the biosynthesis of receptor‐like kinases (RLKs), particularly the lysin motif receptor‐like (LysM‐RLKs) domain proteins (Moellers et al., 2017). Plants’ successful defense against pathogens is dependent on the effectiveness of the first layer, which involves the detection of specific pathogen signatures, also known as pathogen‐associate molecular patterns, and the subsequent induction of appropriate immune response (Chisholm et al., 2006; Jian et al., 2008). LysM‐RLKs are known to be one of the major groups involved in plant defense (Abedi et al., 2021; J. Wan et al., 2008). Specifically, LysM‐RLKs possess molecules that bind to chitin, a main component of fungal cell walls and pathogen's signature molecule upon plant cell invasion (Abedi et al., 2021; J. Wan et al., 2008). Therefore, an immediate and sustained increase in *Glyma.02G059700* expression can be important in maintaining plant defense, thus providing higher levels of resistance as observed in the PR cultivars compared to S cultivars.

*Glyma.09G232100* followed a closely similar expression pattern to *Glyma.02G059700* described above (Table 2; Figure 2). The gene was upregulated in PR cultivar, OAC Drayton, as early as 1 DPI (Fc > 4, *p* < 0.05). However, the earliest significant upregulation for OAC Petrel occurs at 3 DPI with over fivefold increase (*p* < 0.05). Although changes in expression levels were observed in S cultivars, Nattosan and OAC Shire across the DPI, the changes were not significant (*p* > 0.05). The expression levels of *Glyma.09G232100* showed to drop lower in PR cultivars, OAC Drayton and OAC Petrel, than in the S cultivars, Nattosan and OAC Shire, at 5 DPI. However, the drop was not found to be significant (*p* > 0.05) (Table 2; Figure 2).

According to gene annotation and GO biological process description, *Glyma.09G232100* is involved in plant cell wall biogenesis and modification, xylan biosynthesis, and glucuronoxylan metabolic process. J. Wang et al. (2020) have also identified *Glyma.09G232100* among 170 genes in soybean that belong to the pectin methylesterase inhibitors gene family. The plant cell wall is known to be the first line of defense against pathogens. Although the cell wall has often been thought of as a passive protective barrier, several studies have illustrated its active physiological functions during pathogenic attacks (J. Wan et al., 2021). Notably, pectin and xylan, which are some of the main components of the cell wall, have been reported to undergo changes that lead to higher resistance against pathogens such as fungi through cell wall fortification mechanisms (M. J. Hong et al., 2010; J. Wan et al., 2021).

*Glyma.09G232600* was significantly upregulated in the PR cultivar, OAC Drayton (*p* < 0.05) at 1 DPI (Fc > 10, *p* < 0.05), while no significant changes were observed in any of the other three cultivars at both 1 and 3 DPI (*p* > 0.05) (Table 2; Figure 2). Interestingly, the expression levels of *Glyma.09G232600* were found to be reduced by 97% in PR cultivars, OAC Drayton and OAC Petrel, at 5DPI, while no significant reduction was observed in the S cultivars, Nattosan and OAC Shire, at the same timepoint (Table 2; Figure 2).

*Glyma.09G232600* has been narrowly linked to defense response against fungal pathogens, particularly *Fusarium oxysporum* in soybean, the causal agent of sudden death syndrome (Libault et al., 2008). Gene annotation, GO, and biological function analysis also connected *Glyma.09G232600* to defense response against bacterium; however, the main reported functions include chloroplast and photosynthetic activity such as chloroplast relocation, photosynthetic electron transport in photosystem I, photosystem II assembly, and thylakoid membrane organization. Numerous studies have demonstrated the role of chloroplasts in plant defense, particularly through the production and regulation of ROS that may directly kill the pathogen (Lu & Yao, 2018). Connections have also been made between photosynthesis, pathogen infection, and plant defense (Lu & Yao, 2018). Although the described functions above improve the certainty of *Glyma. 09G232600*’s involvement in defense against *S. sclerotiorum*, the wide range of associated processes and inadequate reports, including gene family identity, make it difficult to delineate induced changes in expression levels observed in the current study.

While variable expression patterns were observed for the four candidate genes between PR and S cultivars at 1 and 3 DPI, a shared trend was the downregulation of these genes in both groups at 5 DPI. The specific cause of this downregulation in PR cultivars at 5 DPI of this study requires further investigation. However, previous studies suggest that downregulation of defense genes in resistant cultivars is common in later infection stages. This may be explained by the plant's adaptation to systemic acquired resistance, a long‐term defense strategy that involves downregulation of some local defense genes while activating others systemically (Gao et al., 2015; Luna et al., 2012; Riedlmeier et al., 2017). Additionally, the downregulation may result from the plant's need to reallocate resources from defense to growth and reproduction once the infection has stabilized (Ballaré & Austin, 2019; Fukada, 2024).

This study presents temporal expression changes of selected candidate soybean genes upon *S. sclerotiorum* infection, thus providing an additional layer of information on the molecular‐level interactions that may lead to differential phenotypic responses. However, it is important to note that interactions between plants and pathogens are often too complex, involving numerous individual and collective genes with varying effects. As a result, the precise molecular mechanisms governing resistance or susceptibility can be genotype and context dependent. Therefore, one of the limitations of this study is in the use of a limited number of genes and genotypes used in the experiments, which was within the scope and resources available. To advance the presented work, future studies should aim at increasing the number of genes and genotypes, shed light on gene networks, and establish causal relationships for soybean resistance to *S. sclerotiorum* through comprehensive approaches such as RNA‐Seq, qRT‐PCR, gene knockout, and pathway analysis research.

## 5 CONCLUSION

Elucidating the effect of individual genes underlying QTL for partial resistance to plant diseases is challenging due to the large number of genes with varying effects and complex interplay to produce the phenotype. Previously, Mugabe et al. (2024) reported two QTLs associated with SSR resistance and presented potential candidate genes through genomic analyses on a panel of genetically diverse soybean genotypes. In this study, we aimed to advance the work of Mugabe et al. (2024) by evaluating gene expression changes between PR and S soybean cultivars through qRT‐PCR. All four genes in the study showed significant changes in expression levels between PR and S cultivars and across the different DPIs. The genes have been reported for various roles, including signaling, hormone‐mediated defense pathways, cell wall modification, and response to pathogen signature molecules. These findings suggest that partial resistance to *S. sclerotiorum* may consist of both innate and adaptive immunity strategies. The findings also further corroborate reports on the quantitative nature of partial resistance to *S. sclerotiorum* and highlight the significance of leveraging multi‐approach research to unravel resistance mechanisms on the molecular level. In summary, this study provides candidate genes that serve as a valuable foundation for future studies that can confirm causal resistance and ultimately help create gene‐based molecular markers for breeding SSR‐resistant soybean cultivars.

## AUTHOR CONTRIBUTIONS

**Deus Mugabe**: Conceptualization; data curation; formal analysis; investigation; methodology; resources; validation; visualization; writing—original draft; writing—review and editing. **Mohsen Yoosefzadeh Najafabadi**: Writing—original draft; writing—review and editing. **Christopher Grainger**: Investigation; methodology; resources; validation. **Istvan Rajcan**: Conceptualization; funding acquisition; project administration; resources; supervision; validation; writing—review and editing.

## CONFLICT OF INTEREST STATEMENT

The authors declare no conflicts of interest.

## Data Availability

The raw data supporting the conclusions of this article will be available without undue reservation.
